# Preferential growth of (001)-oriented Bi_2_SiO_5_ thin films deposited on (101)-oriented rutile substrates and their ferroelectric and dielectric properties

**DOI:** 10.1038/s41598-022-19058-y

**Published:** 2022-09-08

**Authors:** Masanori Kodera, Keisuke Ishihama, Takao Shimizu, Hiroshi Funakubo

**Affiliations:** 1 Material Research Center for Element Strategy, Tokyo Institute of Technology, Yokohama, 226-8502 Japan; 2 School of Materials and Chemical Technology, Tokyo Institute of Technology, Yokohama, 226-8502 Japan; 3 Research Center for Functional Materials, National Institute for Materials Science, Tsukuba, 305-0044 Japan; 4 Present Address: Global Zero Emission Research Center, National Institute of Advanced Industrial Science and Technology, Tsukuba, Ibaraki 305-8569 Japan

**Keywords:** Electronic materials, Electronic devices, Electronic devices

## Abstract

Ferroelectric thin films are important because of their great potential for use in various electric devices such as ferroelectric random-access memory. It was expected that Bi_2_SiO_5_, a Si-containing ferroelectric material, would show improved ferroelectricity by targeting a film with the (001)-orientation (polar-axis) on the substrate. Although there was a narrow process window for the deposition of the (010)/(001)-oriented Bi_2_SiO_5_ thin film, it was successfully prepared on a (101)-oriented TiO_2_ single substrate using the pulsed layer deposition technique. The optimum film deposition conditions and film thickness were found, and in this material, the volume fraction of the (001)-oriented domain reached about 70%. By controlling film orientation to the polar axis, the remanent polarization value of this film was 4.8 μC cm^−2^, which is the highest value among reported Bi_2_SiO_5_.

## Introduction

Ferroelectric materials are used in various applications such as ferroelectric random-access memory, actuators, and strain sensors^1–4^. Ferroelectrics exhibit a high dielectric constant (*ε*_r_), stemming from a sharp increase in *ε*_r_ caused by a ferroelectric-dielectric phase transition. Considering the relevance of ferroelectrics to Si-based industries, it is worth investigating ferroelectrics that contain silicon. Despite this, to date, both the number of papers on Si-containing ferroelectric materials and the variety within this class of materials is very limited. Among the Si-containing ferroelectrics, bismuth silicate (Bi_2_SiO_5_, denoted as BSO) is one promising example^5,6^. BSO has a crystal structure analogous to that of Aurivillius-type structures, such as Bi_2_VO_5.5_ (BVO) and Bi_2_WO_6_ (BWO), which are members of the famous ferroelectric family of bismuth layered-structure ferroelectrics (BLSFs). Although BLSFs have a layered perovskite-type structure, in BSO the perovskite-type blocks are replaced by SiO_4_ chains. It was reported that this SiO_4_ chain plays a major role in ferroelectricity and was assumed to exhibit spontaneous polarization of 23.5 μC cm^-2^ along the *c*-axis of BSO^7^. In addition, BSO has recently received attention as a high-dielectric-constant material^8–10^.

Ferroelectric thin films, especially epitaxial films, have the advantage that their ferroelectric and dielectric properties can be controlled, in contrast to the bulk form. This is possible since these properties are greatly affected by the orientation of the film, owing to the asymmetric structure of ferroelectric materials. Among the BLSFs, SrBi_2_Ta_2_O_9_ (SBT) and Bi_4_Ti_3_O_12_ (BIT) epitaxial films have been extensively investigated; these studies have revealed that they exhibit different electrical properties depending on their orientation^11–13^. For these compounds, the film orientation can be controlled by choosing suitable substrates with the correct orientations.

Recently, it was reported that the film orientation of BSO can also be controlled using SrTiO_3_ (STO) substrates with various orientations^14^. The crystal growth of BSO on STO was similar to that of BLSFs on STO substrates because the lattice constants of BSO are close to those of BLSFs, as shown in Table 1. Furthermore, it has been claimed that epitaxial growth is mainly caused by the fluorite-type layers instead of the perovskite-type layers in BLSFs or silicate chains in BSO^15^.

**Table 1:** Lattice constants of BSO and BLSFs. *In-plane O–O length of (101)TiO_2_.

Material		*a* (Å)	*b*(Å)	*c*(Å)	*β* (°)	*b*/*c* in BSO or *b*/*a* ratio in BLSF	References
Bi_2_SiO_5_	(BSO)	15.119	5.444	5.289	90.070	1.032	Kim^7^
Bi_2_VO_5.5_	(BVO)	5.525	5.602	15.27	90	1.014	Coll. Code 85,181
SrBi_2_Ta_2_O_9_	(SBT)	5.531	5.534	24.98	90	1.000	Coll. Code 71,911
Bi_4_Ti_3_O_12_	(BIT)	5.411	5.448	32.83	90	1.007	Coll. Code 16,488
TiO_2_		(5.464)*	(5.464)*	(4.594)*	–	–	Coll. code 9161

Among reported BSO materials, to our best knowledge, the BSO film with the (411)-orientation showed the largest remanent polarization (*P*_r_) of 3.2 μC cm^-2^ in experiments, but further improvement of their ferroelectric properties is required for practical use. Because the major polar axis of BSO is the *c*-axis, it is expected that a (001)-oriented film should exhibit greater ferroelectricity. However, (001)-oriented BSO films have not been reported.

It is often challenging to grow short-axis oriented films in layered structures. In the case of the BLSFs, (100)/(010)-oriented BIT and SBT were successfully grown on (101)-oriented rutile (denoted as (101)TiO_2_) substrates, whereas BVO has not been. The order of the lattice mismatches is likely to be BVO > SBT > BIT. Although the crystal structure of BSO is similar to that of BVO, the lattice parameters of the short axes are similar to those of BIT, as can be seen in Table 1. Therefore, considering the lattice mismatch, BSO is likely to grow on (101)TiO_2_. Moreover, BSO has a large *b*/*c* ratio compared to the BLSFs. It should be noted that the long axes of BSO and the BLSFs are the *a*- and *c*- axes, respectively. Therefore, the ratio of the two short axes is *b*/*c* in BSO and *a*/*b* in the BLSFs. In the case of SBT, the *a*/*b* ratio is almost unity, indicating that preferential growth of (100)- or (010)-oriented films is difficult. However, in the case of BSO, the *b*/*c* ratio is as large as 3%, implying a great potential to control the volume fraction of the (010) and (001)-orientations.

In this study, the deposition conditions for BSO films on (101)-oriented TiO_2_ substrates were investigated. Reciprocal space mapping of X-ray diffraction (RSM-XRD) and high-angle annular dark-field scanning transmission electron microscopy (HAADF-STEM) techniques were used to understand the manner of the crystal growth, which was then compared to that of the BLSFs. Finally, the ferroelectric and dielectric properties of the BSO films were measured to determine the effect of controlling the orientation.

## Experimental section

### Sample preparation

BSO films were prepared by pulsed laser deposition (PLD). The BSO target was synthesized via a solid-state reaction. First, Bi_2_O_3_ and SiO_2_ were mixed in an alumina crucible with a small amount of ethanol for more than 1 h. The molar ratio of Bi/Si was set to 2.5 to compensate for the evaporation of Bi species during the heat treatment. The mixture was then pressed to obtain a pelletized sample. Finally, the pellet was calcined at 1093 K for 10 h to obtain a yellow pellet.

Typically, 0.5 wt% Nb-doped (101)-oriented TiO_2_ (Nb:TiO_2_) substrates were placed on a sample holder using silver paste. The substrates were heated to 823–923 K under a flow of oxygen (3 mL min^-1^) while maintaining the pressure at 280–320 mTorr during the deposition. A 250 mJ KrF excimer laser (*λ* = 248 nm, irradiation area $\approx$ 6 mm^2^) was focused on the rotating BSO target (a pulse repetition rate of 4 Hz) for 10–45 min. After the deposition, the films were cooled without temperature control.

### Characterization

The crystal structures of the films were characterized using X-ray diffraction (XRD, Cu Kα1). RSM was performed using a 2-axis (2*θ*-*ω*) measurement. The film thickness was calculated using wavelength-dispersive X-ray fluorescence (WD-XRF, Axios PW4400/40, PANalytical). HAADF-STEM images were obtained using a JEM-ARM200F microscope (JEOL). Before the electrical measurements, the Pt top electrodes were deposited by electron-beam evaporation. Polarization–electric field (*P*–*E*) hysteresis measurement was performed using a ferroelectric tester (FCE-1A, Toyo Corporation). The *P*_r_ was calculated from the *y*-intercept of the hysteresis loops. The relative dielectric constants and dielectric losses were measured using an impedance analyzer (4194A, Hewlett Packard).

## Results and discussion

**Figure 1:**
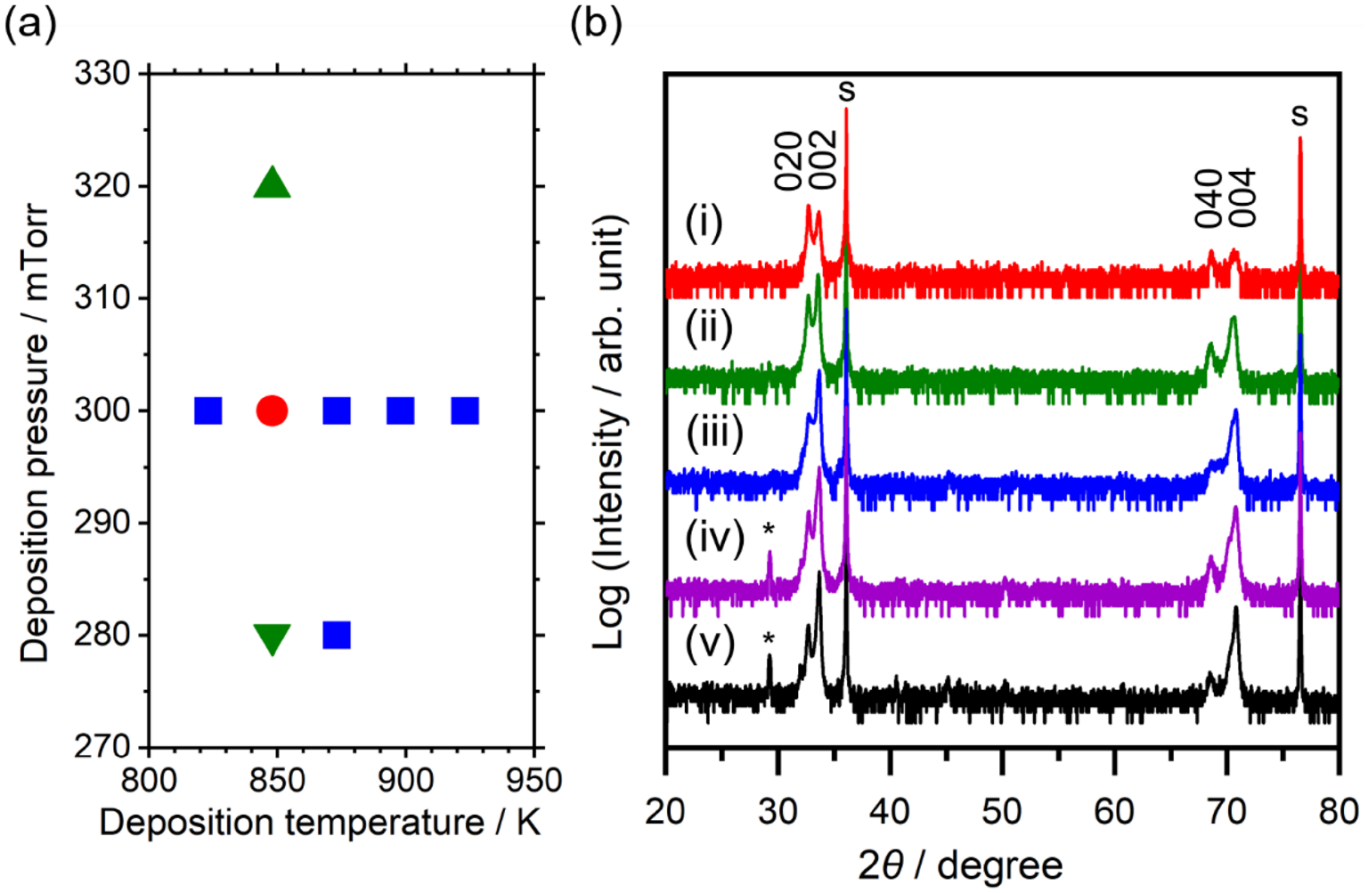
(**a**) Process window for BSO deposition on (101)Nb:TiO_2_. Symbols show as follows. ●: (010)/(001)-orientation, ▲: (010)/(001)-orientation with (411)-orientation, ▼: (010)/(001)-orientation with (411)-and (100)-orientation, ■: (100)-orientation. (**b**) XRD patterns for BSO/(101)Nb:TiO_2_ with various film thicknesses deposited at 848 K and an O_2_ atmosphere of 300 mTorr. (i) 100 nm, (ii) 220 nm, (iii) 300 nm, (iv) 430 nm, and (v) 670 nm. “*” and “s” in the figure indicate peaks attributable to the 311 reflection, and to the substrate, respectively. The X-rays were directed parallel to [010]_TiO2_.

The deposition conditions, such as the temperature, pressure, and time, critically affected the orientation of the BSO films deposited on (101)Nb:TiO_2_ (denoted as BSO/(101)Nb:TiO_2_). The effects of the deposition temperature and pressure on the film orientation are summarized in Fig. 1a. Temperatures higher or lower than 848 K mainly resulted in a (100)-oriented BSO film. Furthermore, a deposition pressure higher or lower than 300 mTorr caused the formation of a small number of (411)-oriented domains. Thus, a very narrow process window was realized. Because BSO has a layered structure, it is reasonable to assume that self-oriented growth along the stacking direction can easily occur. Even under the optimum deposition conditions (at 848 K and 300 mTorr), a small number of (100)- or (411)-oriented domains sometimes formed. Figure 1b shows the XRD patterns for BSO/(101)Nb:TiO_2_ with various thicknesses, all deposited under the optimum conditions. In all of the films, the predominant diffraction peaks belonged to the 0*k*0 and 00* l* classes. Therefore, (010)/(001) was the preferred orientation of the BSO films grown on the (101)Nb:TiO_2_ substrates. In films thicker than 400 nm, small peaks corresponding to the 311 reflections were observed. It has been reported that the 311 reflection is a tail peak from a non-symmetric plane, indicating that (411)-oriented BSO was partially formed in thicker films^16^.

The peak intensity ratio of the 002 to 020 reflections increased as the film thickness increased, and it reached over 20 in the case of the 670 nm-thick film. Therefore, the volume fraction of the (001)-oriented domains apparently increased with increasing film thickness.

**Figure 2:**
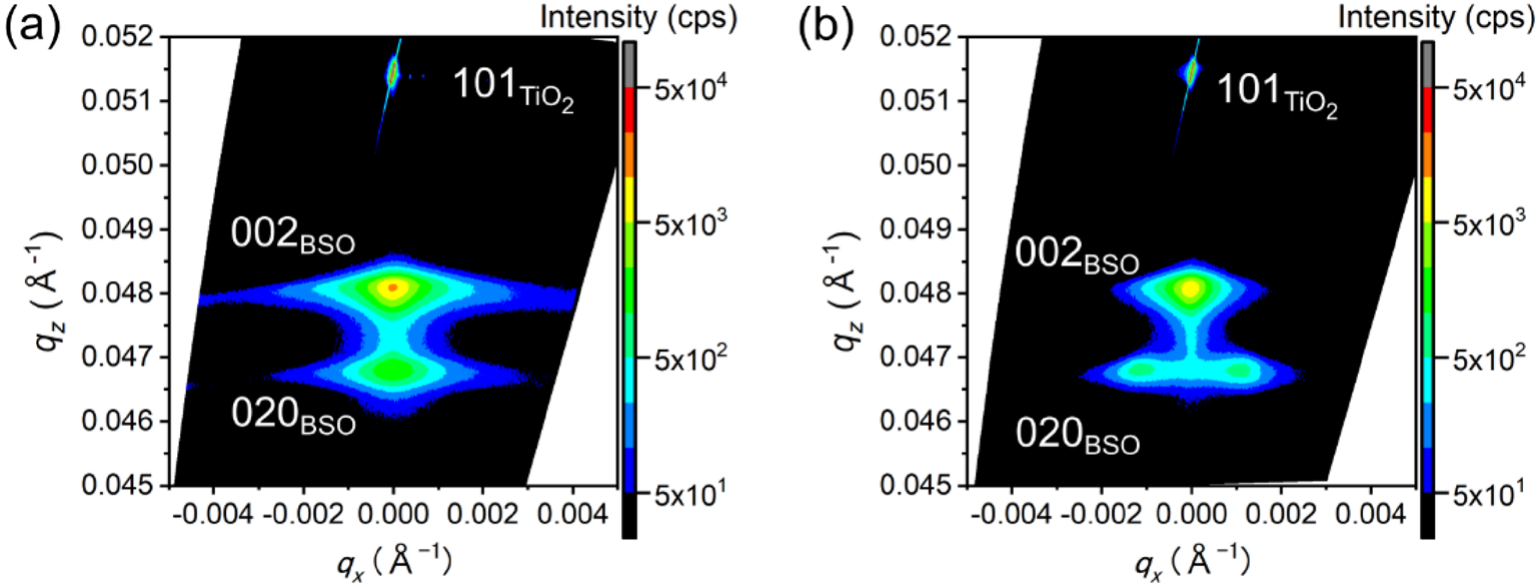
RSMs for BSO/(101)Nb:TiO_2_ with a thickness of 300 nm. X-rays were introduced parallel to (**a**) [10–1]_TiO2_, and (**b**) [010]_TiO2_.

However, in epitaxial films, it is often seen that the tilting of domains reduces the peak intensity in out-of-plane XRD measurements. Therefore, RSM measurements were performed in order to evaluate this effect; the results are shown in Fig. 2. Figure 2a and b show the RSM where the X-rays were directed along the [10–1]_TiO2_ and [010]_TiO2_ directions, respectively. Spots attributable to the 020 reflections were split when measured along the [10–1]_TiO2_ direction, whereas splitting was not observed in Fig. 2a. This result indicates that the (010)-oriented domains tilted along the [10–1]_TiO2_ direction by ± 1.3°.

**Figure 3:**
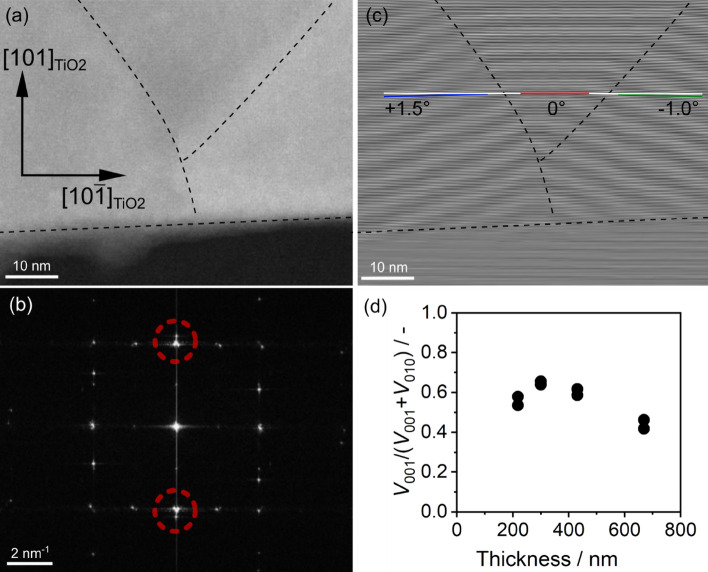
HAADF-STEM for 300 nm-thick BSO/(101)TiO_2_ (**a**) a HAADF-STEM image; (**b**) the FFT image of (**a**); and (**c**) the inverse FFT image reconstructed using the spots marked by red circles in (**b**). (**d**) The thickness dependence on the volume fraction of (001)-oriented domains, calculated from locking curve measurements.

Furthermore, the STEM images in Fig. 3 support the existence of tilted domains. A STEM micrograph of one of the films is shown in Fig. 3a, and a fast Fourier transform (FFT) image derived from this is shown in Fig. 3b. Among the spots in the FFT image, two spots that appeared almost perpendicular to the film growth direction (surrounded by dotted red circles in the figure) were used to reconstruct an inverse FFT image, shown in Fig. 3c. There are three domains present: two tilted domains and one domain without tilting. The absolute value of the tilt angle was 1.0°–1.5°, corresponding to the result in the RSM in Fig. 2b. Interestingly, spot splitting was observed only in the (010)-oriented domain. Although the reason for this is not fully understood, this point is discussed again in the section on crystal growth.

Therefore, the volume ratio of the (001)-oriented domain (V_001_) to the (010)-oriented domain (V_010_) was estimated using the RSM or XRD rocking curve measurements. The dependence of the film thickness on the calculated volume ratio of the (001)-oriented domain (*V*_001_/(*V*_001_ + *V*_010_)) is plotted in Fig. 3d. It should be noted that the expected intensity ratio of the 002 reflection to the 020 reflection from the polycrystalline powder (random orientation) was assumed to be 1.23, which was estimated using the BSO structure reported by Kim et al.^7^ The value of *V*_001_ decreased for films with thicknesses greater than 400 nm. Considering that a small number of (411)-oriented domains are also present in films thicker than 400 nm, the volume fraction of the (001)-oriented domain in the film should be even smaller. Because the lattice mismatch between the *d*_101_ of TiO_2_ and the *d*_010_ of BSO is expected to be smaller than that of the *d*_001_ of BSO, as shown in Table 1, there was preferential growth of the film in the (001)-orientation. Generally, in thicker films the strain induced by lattice mismatch is reduced due to the introduction of defects, and the volume fraction of the (001)-oriented domains decreases. The author did not fully understand the lower volume fraction of (001)-domain in the thinner region such as 200 nm-thick. XRD results showed the thinner film showed lower crystallinity and STEM images indicated there were defects at the interface between the film and the substrates, suggesting that the relatively large defect density at the interface should reduce strain.

**Figure 4:**
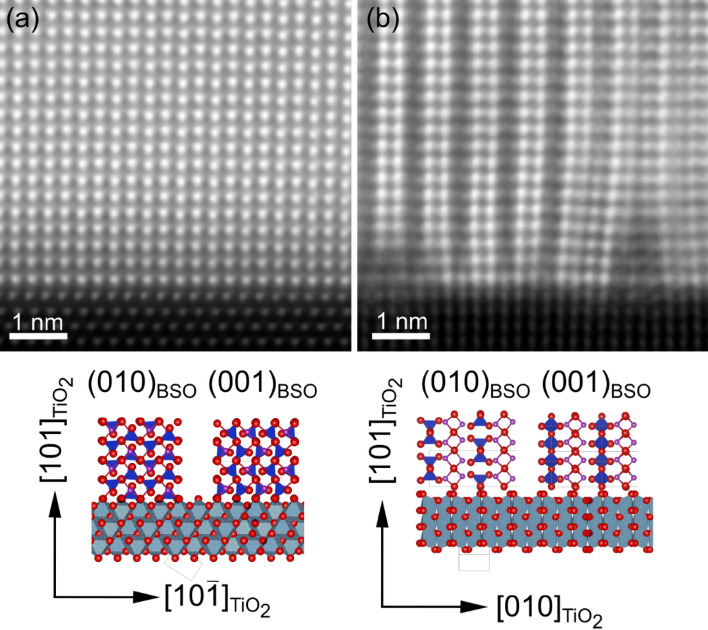
HAADF-STEM images for BSO/(101)Nb:TiO_2_ with a 300 nm thick film, viewed from (**a**) the [010]_TiO2_, and (**b**) the [10–1]_TiO2_ directions.

To study the interface between the BSO and the Nb:TiO_2_, HAADF-STEM images were obtained, as shown in Fig. 4. In Fig. 4a, the bright spots indicate Bi ions, indicating coherent growth on the TiO_2_ units. Therefore, heteroepitaxial growth was confirmed for the (010)/(001)BSO/(101)TiO_2_ system. Furthermore, this result indicates that the fluorite-type layers play a major role in heteroepitaxial growth. A sharp interface without any interlayers between the film and the substrate was observed. This is clearly different from (411) BSO/(110)STO, in which there is an interlayer. On the other hand, when observed in the [10–1]_TiO2_ direction, discontinuous layers were found at the interface between the film and the substrate (Fig. 4b). The O–O distance between two fluorite-type layers of the BSO is 7.47 Å, whereas 2 × *d*_010_ for the TiO_2_ substrate is 9.19 Å. This represents a mismatch of over 20%, which is the cause of the film defects.

**Figure 5:**
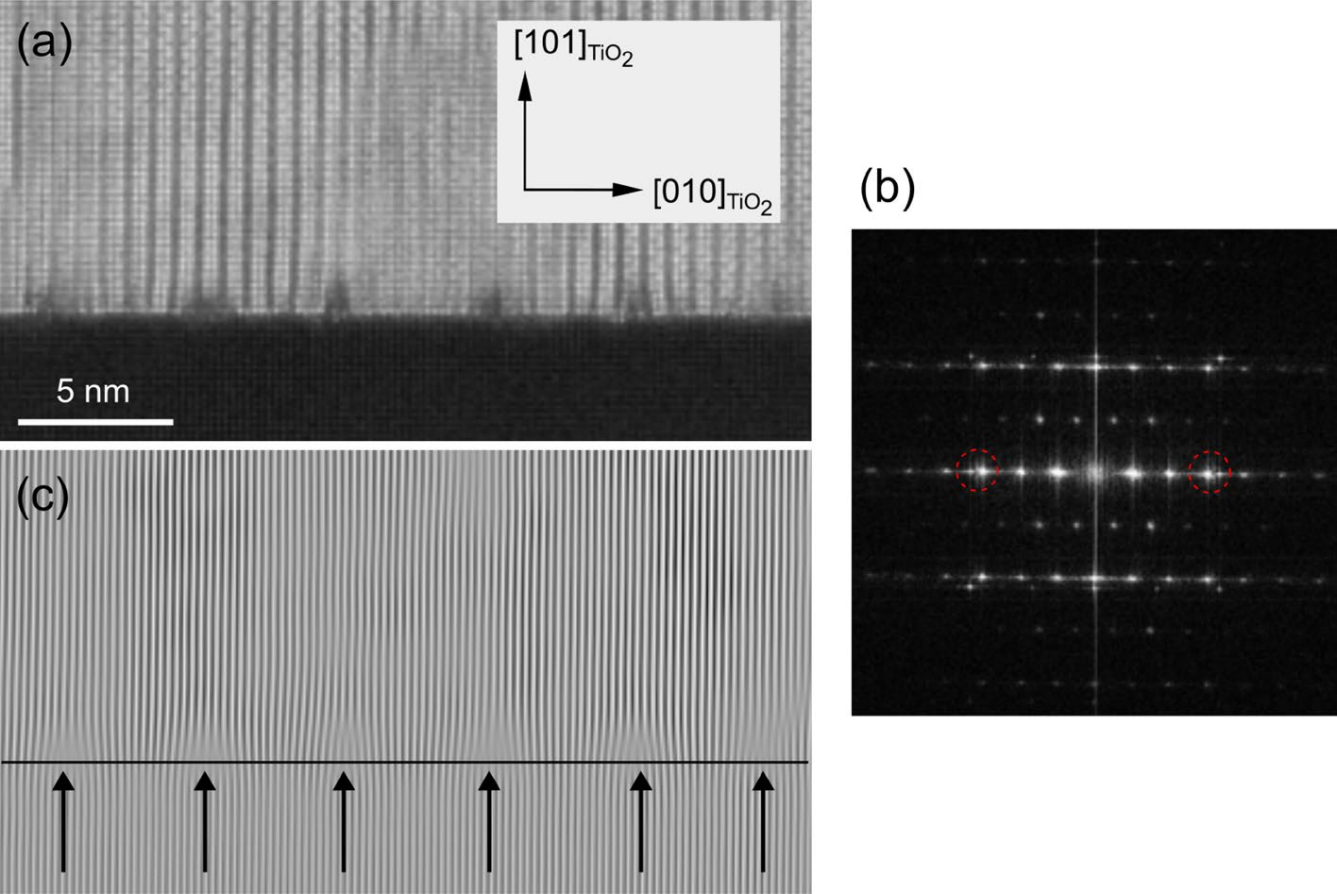
HAADF-STEM images of BSO/(101)Nb:TiO_2_: (**a**) a micrograph, and (**b**) its FFT image. (**c**) The inverse FFT image, which was reconstructed using 020_TiO2_ and 600_BSO_ [spots marked by red circles in (**b**)]. The arrows in (**c**) indicate the defects.

Figure 5a presents HAADF-STEM images from a wider angle than that of Fig. 4b. In Fig. 5c, is an inverse FFT image that was constructed using 020_TiO2_ and 600_BSO_, which are marked by red circles in Fig. 5b. It can clearly be seen that the defects were introduced into the BSO films at the interface. On average, a discontinuous layer formed for every six to seven fluorite-type layers. This trend is similar to that observed for BIT/(101)TiO_2_. In this case, the long axis of BIT matched well with the *d*_010_ of TiO_2_. One unit of BIT had almost the same length as seven rutile units, which corresponded to a 2.8% mismatch on average. Moreover, in the case of BSO/(101)TiO_2_, the distorted areas near the defects appear to be larger than those of BIT/(101)TiO_2_. As a result, the BSO film has a larger defect density than the BIT film.

In Bi_2_SiO_5_ thin films, little is known about domain structure. Therefore, it is worthwhile to analyze domain structures. XRD results in Fig. 2b and TEM images in Fig. 3c clearly showed the tilting of domains, indicating forming of elastic domains. In Fig. S1, cross-sectional HAADF-STEM images of BSO/(101)Nb:TiO_2_ were presented. Two kinds of domains, (010)-oriented domain (*b*-domain) and (001)-oriented domain (*c*-domain) were observed. Domain size was at least several tens of nanometers in Fig. S1b. In Fig. S1a, the domain boundary between the *c*-domain and *b*-domain was smooth and parallel to the substrate while the domain boundary between *b*- and *c*-domain in Fig. S1b was tilting. It is known that phase transition from paraelectric orthorhombic phase to ferroelectric monoclinic phase occurs around 663 K in a single crystal^5^. Although the deposition temperature of BSO film was above Curie temperature of BSO, domain boundary was considered to form during the deposition because *b*- and *c*-domain in the orthorhombic phase should be kept after phase transition.

A brief summary of the crystal growth in the BSO/(101)Nb:TiO_2_ system is presented here. A narrow process window was discovered for the successful growth of BSO films with the (010)/(001)orientation. Along the *a*-axis, a large number of defects were introduced at the interface between the film and the substrate. On the other hand, the *b*- and *c*-axes of BSO matched well with the *d* values of TiO_2_, and hence grew coherently. Because the lattice mismatch between *d*_101_ of TiO_2_ and the *d*_010_ of BSO is smaller than its mismatch with the *d*_001_ of BSO, the film grew preferentially in a (001)-oriented domain. Furthermore, when (010)BSO formed, in-plane tensile strain was induced in the (010)-oriented domain. Lattice relaxation occurred when the film thickness increased, probably triggering the domain tilting. This phenomenon is analogous to the *a*/*c*-domain formation in the PbTiO_3_/STO system^17–19^. In the PbTiO_3_/STO case, the domain boundary between the (100)- and (001)- domains was 45°, which is similar to the result in Fig. 3a.

**Figure 6:**
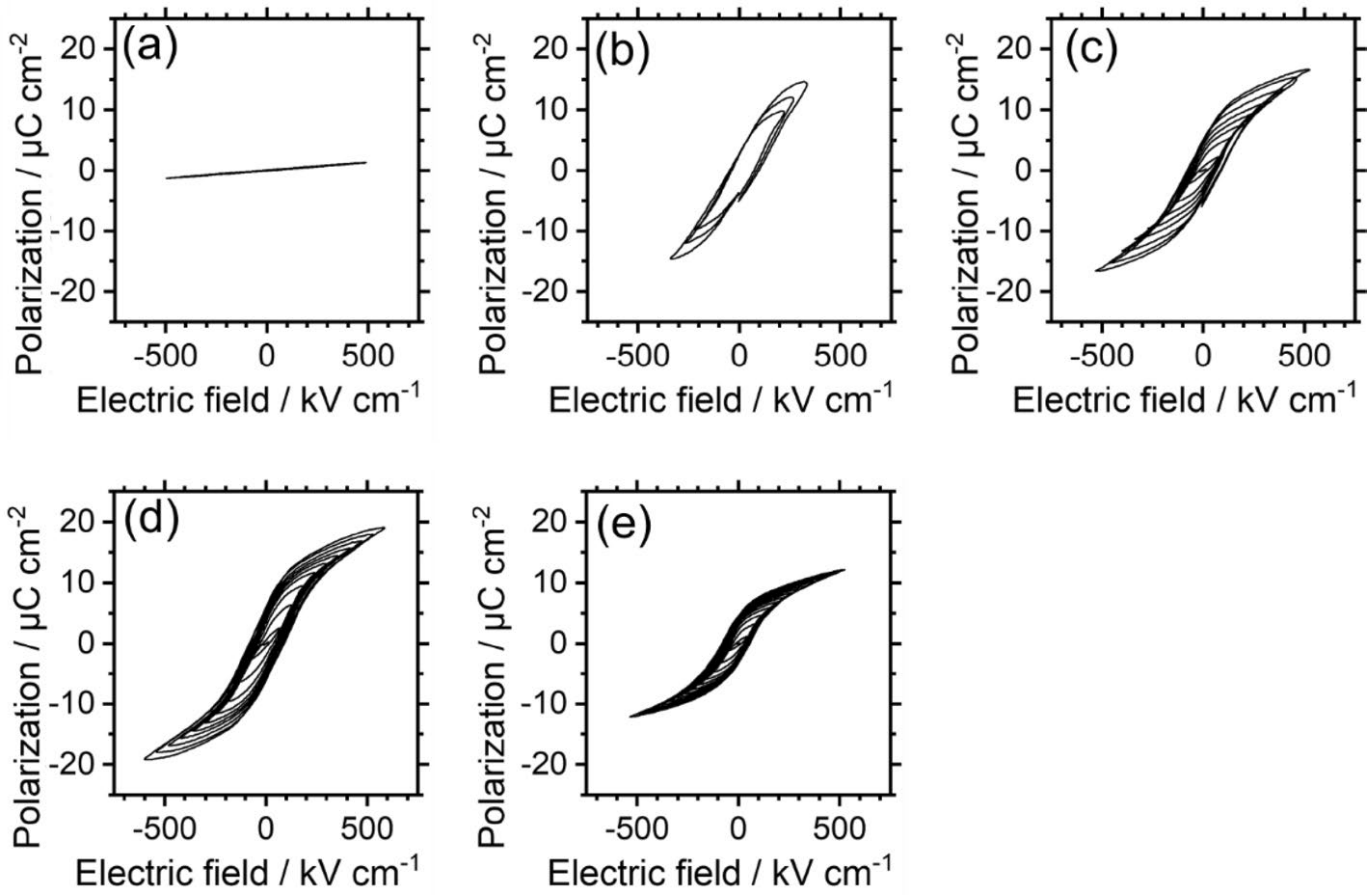
Polarization–Electric field loops for BSO/(101)Nb:TiO_2_ with various film thicknesses: (**a**) 100 nm, (**b**) 220 nm, (**c**) 300 nm, (**d**) 430 nm, and (**e**) 670 nm.

Figure 6 shows the thickness dependence of the *P*-*E* hysteresis loops for BSO/(101)Nb:TiO_2_. In the case of the 100 nm-thick BSO film, no hysteresis based on ferroelectricity was observed. In contrast, clear hysteresis was observed in films thicker than 200 nm. The remanent polarization (*P*_r_) reached a maximum value of about 4.8 μC cm^-2^ for the 300 nm-thick film, but then decreased as the films got thicker. To our best knowledge, (411)BSO/(110)Nb:STO exhibited the largest *P*_r_ value among reported BSO materials, at 3.2 μC cm^-2^. Therefore, the successful control of the BSO film orientation that was employed in this study increased the ferroelectric properties of the BSO film by a factor of 1.5. When the films are thicker than 400 nm, the (010)- and (411)-oriented domains increase, resulting in smaller remanent polarization values. Despite the high *P*_r_ value attained in this study, it is still only one-fifth of the value estimated by Kim et al.^7^ One possible reason for this is that the large defect density likely increased the leakage current, resulting in reduced ferroelectric properties. The insulation property is shown in Fig. S2, supporting the above discussion.

Finally, the dielectric constant and dielectric loss of BSO/(101)Nb:TiO_2_ films with various thicknesses were measured. Figure S3a presents the results for a 300 nm-thick sample, and the thickness dependence of the dielectric properties at 100 kHz is summarized in Fig. S3b. For all film thicknesses, the dielectric loss is smaller than 0.08. For the case of the 100 nm-thick film, a relatively small dielectric constant was measured, probably owing to the contribution of the interface layer. The largest dielectric constant, of approximately 150, was obtained for the 200 nm-thick sample. This can be compared to the reported dielectric constants for other BSO films, such as (100)-, (411)-, and (201)/(210)-oriented BSO/STO, which exhibit relative dielectric constants of 20, 65, and 100, respectively^16^. The XRD analysis in the present work revealed that some (411)BSO formed in the thicker films, which resulted in reduced dielectric constants; this may explain the trend seen in Fig. S3b.

Although both the ferroelectric and dielectric properties of BSO films can be enhanced by controlling the film orientation, there is still much room for improvement. This can be achieved by further optimizing the deposition conditions such as the target composition, and optimizing the bottom electrodes. It is known that the conductivity of Nb-doped substrates is not high compared to Pt or conductive oxides such as SrRuO_3_ and LaNiO_3_, leading reduction of effective applied voltage. One promising strategy is to further optimize the substrate in order to reduce the mismatch of the lattice and the *a*-axis of the BSO. For example, a potential method to achieve this is to introduce interlayers, such as IrO_2_, RuO_2_ on TiO_2_. Another possible approach is the doping or substitution of different elements into BSO. This may affect other properties, such as dielectric constants and leakage properties, in addition to the lattice constants.

## Conclusion

The (010)/(001)-oriented BSO film was successfully deposited onto a (101)Nb:TiO_2_ substrate. A narrow process window in terms of the deposition temperature and pressure was determined. Among the obtained films, the volume fraction for the (001)-oriented domain reached a maximum value of approximately 70% at a thickness of 300 nm, which proved the preferential growth of the (001)-oriented domain. Tensile strain induced in the (010)-oriented domain may have caused it to be tilted.

At the interface between the BSO film and the substrate, defects were observed in a regular pattern along the *a*-axis of BSO, while coherent growth was observed along the *b*- and *c*-axes. The fluorite-type layer was found to play a major role in heteroepitaxial growth, which is in good agreement with the reported results for (411)BSO/(110)STO. The tilting of domains and domain boundaries between *b*-domain and *c*-domain clearly showed that BSO formed some elastic domain structures while further investigation is needed. Both the remanent polarization and dielectric constants were improved compared to the reported epitaxial BSO films owing to the orientation control. The obtained *P*_r_ value of 4.8 μC cm^-2^ is the highest among the reported values for Bi_2_SiO_5_ thin films. Reducing the lattice mismatch further should reduce the defect density at the interface between the film and the substrate, which should improve the ferroelectricity. Our study not only experimentally shows the potential of BSO films and Si-containing ferroelectrics but also provides beneficial information about the mechanism of heteroepitaxial growth where the film and the substrate have different crystal structures.

## Supplementary Information

Supplementary Information.

## Data Availability

The datasets generated during and/or analyzed during the current study are available from the corresponding author on reasonable request.
