# Supplementary material for: Preferential growth of (001)-oriented Bi2SiO5 thin films deposited on (101)-oriented rutile substrates and their ferroelectric and dielectric properties
Source: Sci Rep. 2022 Sep 8;12:15204. doi: 10.1038/s41598-022-19058-y (PMC9458711; doi:10.1038/s41598-022-19058-y)
Supplement: Supplementary file 1 — Supplementary Information. [file 41598_2022_19058_MOESM1_ESM.pdf]

Supporting Information

Preferential growth of (001)-oriented Bi_2SiO_5 thin films deposited on (101)-oriented rutile substrates and their ferroelectric and dielectric properties

Masanori Kodera[1*†], Keisuke Ishihama[2], Takao Shimizu[2,3], and Hiroshi Funakubo[1,2*]

[1] *Material Research Center for Element Strategy, Tokyo Institute of Technology, Yokohama, 226-8502, Japan*

[2] *School of Materials and Chemical Technology, Tokyo Institute of Technology, Yokohama 226-8502, Japan*

[3] *Research Center for Functional Materials, National Institute for Materials Science, Tsukuba, 305-0044, Japan.*

[†] Present address: Global Zero Emission Research Center, National Institute of Advanced Industrial Science and Technology, Tsukuba, Ibaraki, 305-8569, Japan

Keywords: Bi_2SiO_5, pulsed laser deposition, epitaxial thin films, ferroelectrics, crystal growth

Contents:

Fig. S1. HAADF-STEM images for BSO/(101)Nb:TiO_2 with a 300 nm thick film.

Fig. S2. Insulation property of 400 nm-thick BSO/(101)Nb:TiO_2.

Fig. S3. Frequency dependence of the relative dielectric constant and dielectric loss (tanδ).

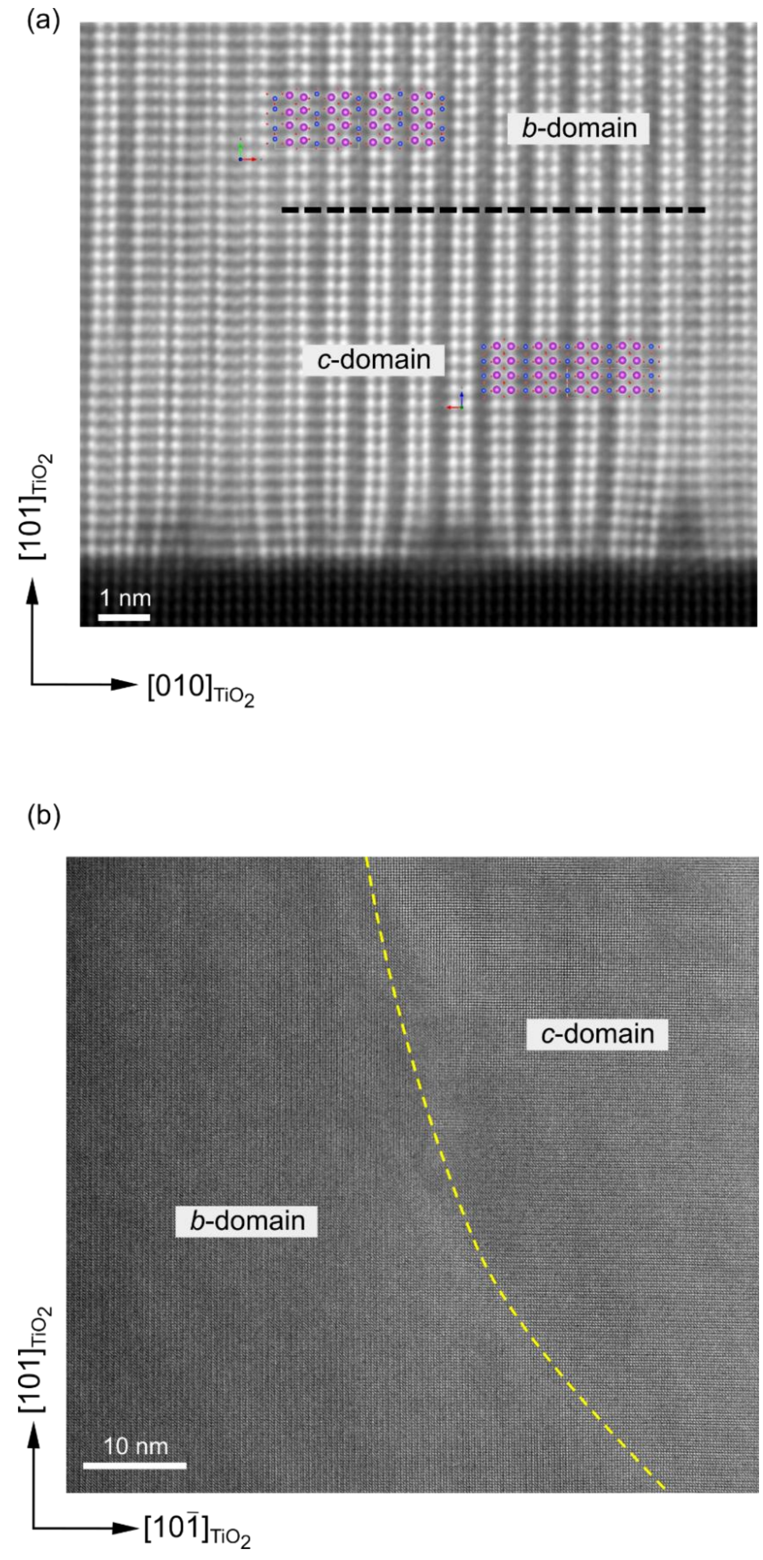

Fig. S1. HAADF-STEM images for BSO/(101)Nb:TiO_2 with a 300 nm thick film, viewed from (a) the [10-1]$_{TiO2}$, and (b) the [010]$_{TiO2}$ directions. Dashed lines indicate domain boundary between *b*-domain and *c*-domain.

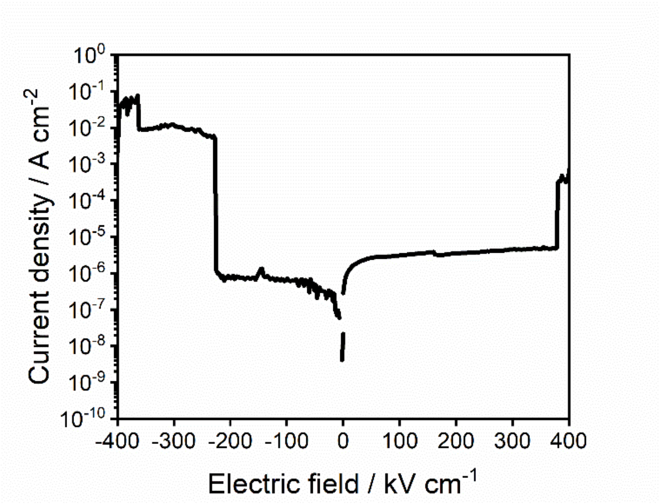

Fig. S2. Insulation property of 400 nm-thick BSO/(101)Nb:TiO_2.

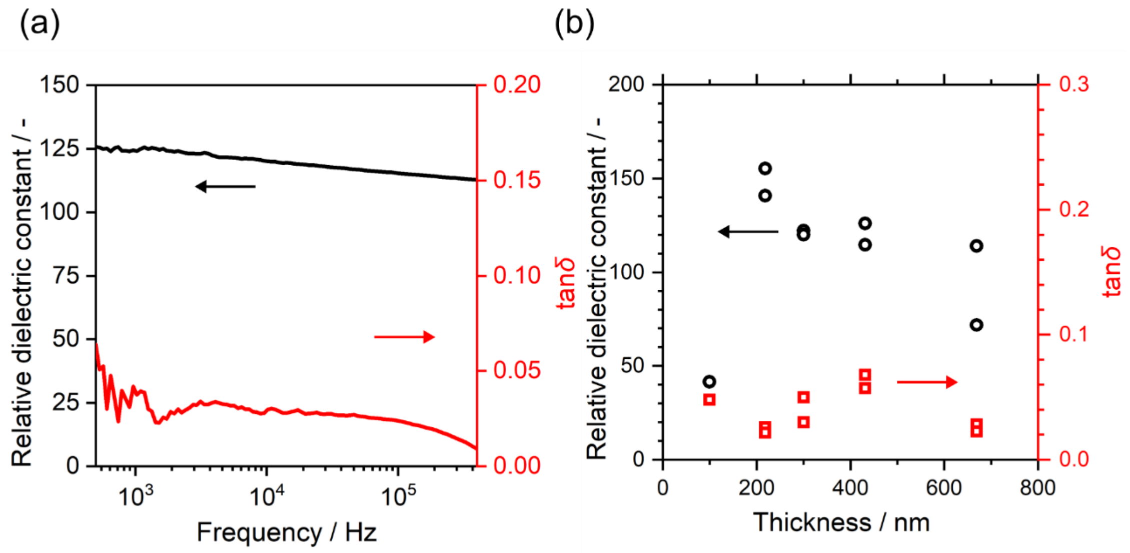

Fig. S3. (a) Frequency dependence of the relative dielectric constant and dielectric loss (tanδ) of 300 nm-thick BSO/(101)Nb:TiO_2, and (b) relative dielectric constants and dielectric loss at 100 kHz for BSO/(101)Nb:TiO_2 with various film thicknesses.
